# Vagal Splenic-Dependent Effects Influence Glucose Homeostasis, Insulin Secretion, and Histopathology of the Endocrine Pancreas in Hypothalamic Obese Male Rats: Vagus Nerve and Spleen Interactions Affect the Endocrine Pancreas

**DOI:** 10.1155/tswj/9910997

**Published:** 2025-04-17

**Authors:** Bruna Schumaker Siqueira, Ellen Carolina Zawoski Gomes, Thiago Rentz, Ananda Malta, Paulo Cezar de Freitas Mathias, Sandra Lucinei Balbo, Sabrina Grassiolli

**Affiliations:** ^1^Programa de Pós-Graduação em Biociências e Saúde, Universidade Estadual do Oeste do Paraná-Campus de Cascavel, Cascavel, Brazil; ^2^Centro Universitario Fundacao Assis Gurgacz, Cascavel, Brazil; ^3^Brigham and Women's Hospital, Boston, Massachusetts, USA; ^4^Universidade Estadual de Maringa, Maringá, Brazil

**Keywords:** islets, obesity, parasympathetic nervous system, spleen

## Abstract

Vagus nerve (VN) and spleen dysfunctions are often associated with obesity (Ob).

**Aim:** We evaluated the effects of VN and spleen ablation on adiposity, metabolism, and insulin secretion in hypothalamic obese male rats.

**Methods:** Ob was induced by neonatal subcutaneous injection of monosodium glutamate (4 g/kg). At 60 days of life, Ob animals were randomly distributed into four groups (*n* = 16 rats/group): sham operation (SHAM), vagotomy (VAG), splenectomy (SPL), and VAG + SPL. Body weight and food intake were monitored for 8 weeks postsurgery. Intraperitoneal glucose tolerance test (ipGTT) and intraperitoneal pyruvate tolerance test (ipPTT) were performed at 148 days of life, and VN activity was recorded at 150 days. After euthanasia (150 days), adiposity, plasma biochemical parameters, glucose-induced insulin secretion (GIIS), and cholinergic and adrenergic islet responsiveness were evaluated. The pancreas was submitted for histopathological analysis, and the protein content of OXPHOS and IL-10 was evaluated in isolated pancreatic islets.

**Results:** Decreased VN activity was confirmed in the Ob-VAG groups, associated with lower visceral adiposity, triglycerides, and plasma insulin, together with improved insulin sensibility and pyruvate tolerance, compared to Ob-SHAM rats. Spleen absence reduced VN activity and cholinergic insulinotropic responses, with deleterious effects on the endocrine pancreas. Furthermore, Ob-VAG + SPL rats presented greater reductions in GIIS and more severe endocrine pancreas histopathology, compared to the Ob-SHAM group, without altered islet size or number or protein content of OXPHOS or IL-10.

**Conclusion:** Vagal and splenic interactions contribute to glucose homeostasis control in hypothalamic obese rats, modulating insulin secretion and pancreas histology.

**Summary**

• Hypothalamic obese rats present greater adiposity and insulin resistance and increased collagen deposition within the pancreatic islets.
• Vagus nerve ablation exerts more antiadiposity effects in hypothalamic obesity than splenectomy.
• Vagal and splenic interactions regulate glucose homeostasis in hypothalamic obesity.
• Spleen absence modulates vagus nerve activity in hypothalamic obese rats.
• The vagus nerve influences collagen deposition in the pancreatic islets of obese hypothalamic rats.
• Vagal and splenic interactions can influence the process of lipomatosis and vacuolization in the pancreas of hypothalamic obese rats.
• Spleen ablation increases leukocyte infiltration in the endocrine pancreas of hypothalamic obese rats.

## 1. Introduction

Healthy body weight (BW) composition results from a proper balance between caloric intake and energy output, a process tightly regulated by hormonal and neural mechanisms, with the autonomic nervous system (ANS) playing a central role [1]. The ANS also modulates immune responses. This is especially relevant in obesity, where a state of low-grade chronic inflammation directly contributes to insulin resistance (IR) and disrupts glucose and lipid homeostasis [2, 3].

In this context, an imbalance in energy metabolism promotes excessive expansion of white adipose tissue (WAT), disrupting the production of adipokines and cytokines, which sustains a systemic proinflammatory state, characterized by elevated circulating levels of tumor necrosis factor-alpha (TNF-*α*), interleukin 1 (IL-1), and interleukin 6 (IL-6), along with reduced levels of anti-inflammatory cytokines such as adiponectin and IL-10 [4, 5].

The parasympathetic autonomic division is pivotal in mediating regulatory interactions between metabolism and neuroimmune response. As the primary pathway for parasympathetic input, the vagus nerve (VN) modulates the function of key endocrine and immune organs, including the pancreas and spleen [6–8].

In the endocrine pancreas, vagal hyperactivity increases the insulinotropic and proliferative effects of acetylcholine (Ach), leading to hyperinsulinemia [9–11]. Increased insulin levels, due to adipogenic and lipogenic insulin's actions, promote the expansion of WAT mass [12, 13]. Furthermore, hyperinsulinemia contributes to IR and results in excessive beta cell activity, factors that in the long term induce beta cell stress and apoptosis [14]. Consequently, VN ablation (vagotomy) in humans and obese rodent models often reduces WAT, restores insulin response, and reestablishes glucose and lipid homeostasis [15–18].

The VN activity, through the cholinergic anti-inflammatory reflex, regulates cytokine production, a process that appears to be spleen-dependent [7, 19, 20]. In this context, although the VN does not directly innervate splenocytes, cholinergic preganglionic vagal neurons—at the level of the celiac ganglion—can stimulate postganglionic sympathetic neurons (splenic nerve), leading to the release of norepinephrine, which suppresses TNF-*α* production by splenic macrophages. According to this hypothesis, norepinephrine acts directly on splenic macrophages stimulating beta-adrenergic receptors, thereby reducing TNF-*α*. Moreover, norepinephrine can stimulate Ach-synthesizing T lymphocytes, which through alpha 7 nicotinic receptor (*α*7nAChR) on splenic macrophages also reduce TNF-*α* [21].

Thus, during inflammatory conditions, the afferent arm of the VN detects peripheral inflammation and relays signals to the brain, which in turn dampens the disease response by activating anti-inflammatory vagal reflex, leading to cholinergic-mediated immunosuppressive splenic responses. Consistent with this, electrical stimulation of the VN may reduce inflammation in various disease conditions [2, 22].

Interestingly, studies have demonstrated that failure in the anti-inflammatory vagal reflex may contribute to the proinflammatory state and metabolic dysregulation found in obesity and diabetic state [8, 23]. In addition to its role in anti-inflammatory vagal reflex, the spleen also influences metabolism and adiposity. Obesity induced by high-fat diet results in heightened inflammation in the pancreas, liver, and WAT when the spleen is absent; this response appears to be dependent of anti-inflammatory IL-10 derived from splenic tissue [24, 25].

We have demonstrated that the spleen can modulate WAT content, glucose tolerance, and glucose-induced insulin secretion (GIIS) in isolated pancreatic islets from lean and obese rats [26, 27]. Furthermore, in healthy rats, we have shown that interactions between VN and the spleen can influence insulin secretion and IL-1*β* content in the endocrine pancreas, as well as alter IL-10 plasma levels and vagal activity [28], a response that is unknown in obesity.

Monosodium glutamate (MSG)–induced obesity is a well-established experimental model characterized by hypothalamic injury, particularly in the arcuate nucleus (ARC) [29]. These hypothalamic lesions lead to extensive WAT expansion, hyperinsulinemia, glucose intolerance, and dyslipidemia, which are often associated with VN hyperactivity and splenic alterations [17, 30–32]. Vagotomy in hypothalamic obese rodents has been shown to normalize insulin plasma levels, reduce WAT accumulation, and improve glucose and lipid homeostasis [17, 33]. Likewise, splenectomy has been reported to elicit similar metabolic benefits in this model [26, 27, 34]. However, whether VN–spleen interactions significantly affect metabolic adaptations to hypothalamic obese rats remains poorly understood. Thus, in this study, we investigated the impact of VN and spleen ablation on adiposity, glucose homeostasis, and endocrine pancreas histology in hypothalamic obese male rats.

## 2. Material and Methods

### 2.1. Ethical Aspects

Animals were kept in cages (three rats/cage) and received water and free access to rodent chow (Biobase, Biotec, Águas Frias, Santa Catarina, Brazil) ad libitum, under controlled conditions of light (12:00–12:00 light–dark) and temperature (23°C ± 2°C), as recommended by the guidelines of the National Council for the Control of Animal Experimentation (CONCEA) and the Arrive Guidelines 2.0. The Committee for Ethics in the Use of Animals (CEUA) of State University of Western Parana (UNIOESTE) approved all experimental protocols on March 16, 2017.

### 2.2. Animals

Pregnant Wistar rats were housed in individual cages with ad libitum access to water and rodent chow until they gave birth. After the first live day, the litter size was adjusted to 6–8 male pups per dam to prevent changes in caloric intake during lactation. Hypothalamic obesity was induced through chemical damage to the ARC, using high doses of MSG, as proposed by Scallet and Olney [35], with adaptations [27]. Thus, from the second to sixth postnatal days, male Ob pups received subcutaneous high doses of MSG (4 g/kg of BW), while the nonobese or CTL group received an equimolar saline solution (NaCl, 1.25 g/kg). All animals were weaned at 21 days of life.

### 2.3. Surgical Procedures

At 60 days of life, the Ob group was randomly subdivided into four subgroups, according to surgical procedures. The VAG procedures were performed according to the protocols of Kiba et al. [36] with adaptations [12]. Briefly, after fasting (12 h), the animals were anesthetized with isoflurane (1%) under spontaneous ventilation with an oxygen mask (1 mL/min). Next, an incision (±2 cm) was made just below the sternum, the esophagus was localized, and, with the aid of a magnifying glass, the anterior and posterior branches of the VN were identified, isolated from the esophageal wall, and tied with surgical thread for sectioning. The ablation of the spleen was performed according to Leite et al. [27]. For this, after anesthesia, the spleen was found and exteriorized from the abdominal cavity; the vessels were tied with cotton thread and sectioned to remove the organ. In the VAG + SPL group, both surgical techniques were performed simultaneously.

The SHAM groups were submitted to anesthesia and exploratory laparotomy with manipulation of the viscera but without any intervention. Thus, considering surgical procedures, Ob-hypothalamic animals formed four groups: Ob-SHAM, Ob-SPL, Ob-VAG, and Ob-VAG + SPL (*n* = 16 male rats for group). CTL-SHAM and Ob-SHAM groups were then compared to evaluate hypothalamic obesity characteristics, and effects of VAG and/or SPL were evaluated in Ob animals. After the surgical procedure, all animals were housed in individual cages, with free access to food and water, for 7 days, to ensure adequate recovery. Afterwards, according to the subgroups, the animals were relocated into groups (3–4 rats/cage).

### 2.4. BW and Evolution of Food Consumption

At 70 days of life, each Ob rat received an individual tail tag, and its food intake and BW evolution were recorded weekly until 145 days of life. To obtain individual food consumption, the following formula was used: [(total feed consumed per cage (g)/number of animals per cage/individual BW)∗100]. These data were used to elaborate area under the curve (AUC) of food intake and BW evolution. Additionally, the BW gain (ΔBW) was obtained from the difference in BW at 145 days minus BW at 70 days of life. The ΔBW and food intake values were used to obtain feed efficiency (FE), as proposed by Duivenvoorden et al. [37], where FE = [ΔBW (g)/∑food intake (g)∗100].

### 2.5. Intraperitoneal Glucose Tolerance Test (ipGTT) and Intraperitoneal Pyruvate Tolerance Test (ipPTT)

At 148 days of life, and after fasting for 12 h, the Ob rats were submitted to ipGTT or ipPTT. The animals were placed in individual cages, a blood sample was collected from the tail vein, and fasting glycemia was evaluated using a glucometer (Accu-Chek Active, Roche Diabetes Care, Jaguaré, Brazil). Subsequently, glucose (2 g/kg of BW) or pyruvate (1 g/kg of BW) was administered intraperitoneally, and glucose levels were evaluated at 15, 30, 60, and 120 min, after overload. The glycemic levels obtained from the ipGTT and ipPTT were used to calculate respective AUC.

### 2.6. Euthanasia, Adiposity, and Stomach Weight

At 150 days of life and after 12 h of fasting, the animals in all groups were weighed, briefly desensitized with CO_2_, and the nasoanal length (NAL, centimeter) was immediately recorded before the animal was decapitated. Obesity was estimated by Lee's index formula (LI): [$\sqrt[{3}]{\text{BW}}\left(\text{g}\right)/\text{NAL }\left(\text{cm}\right)$] [38]. Blood was collected in tubes and serum was used for metabolic and hormonal analyses, as described in the next subitem. The abdominal cavity was opened, and the pancreas was excised, weighed, and subjected to histological analysis or islet isolation, according to the procedure described below. Adiposity was evaluated by weight of WAT, excised from subcutaneous inguinal (I) and visceral mesenteric (M) depots. Gastric stasis results from VAG, causing an increase in stomach weight [39]. Therefore, immediately after euthanasia, the stomach was excised, opened, cleaned, and weighed.

### 2.7. Serum Biochemistry Analysis and Insulin and Interleukin-10 Levels

Glucose, triglycerides, and total cholesterol were measured in serum, using enzymatic/colorimetric kits (Bioloquid, Laborclin Produtos para Laboratórios Ltda, Pinhais, Brazil), and insulin was measured by radioimmunoassay (RIA). Serum IL-10 levels were assessed using ELISA (Novex, Life Technologies, KRC0101, KRC0102). Fasting glucose and triglyceride values were used to assess IR by the TyG index, using the formula [Ln (fasting triglycerides mg/dL)∗(fasting glucose mg/dL)/2] [40].

### 2.8. Islet Isolation, Insulin Secretion, and Protein Expression

Pancreatic islets were isolated from 5 to 6 rats per group, using the collagenase technique [41] with modifications [42] Briefly, after abdominal laparotomy, the hepatic common bile duct was found and the pancreas was perfused with Hank's buffer solution (HBS) [pH 7.4, bovine serum albumin (BSA, 0.1%); 5.6 mM glucose; 95/5% oxygenated (O_2_/CO_2_)] containing collagenase type V (1 mg/mL). The organ was excised at once and transferred to a falcon tube containing 10 mL of HBS solution at 37°C for approximately 15 min, for the collagenase to act. The tissue mass was filtered, and the islets were isolated and transferred (4 islets/well) to a 16-well plate containing 1 mL of Krebs buffer solution (KBS) [pH 7.4, 0.1% BSA and 5.6 mM glucose] in a gassed condition (CO_2_, 5%), at 37°C for 60 min, during the preincubation phase. Afterwards, the supernatant was discarded and 1 mL of KBS (pH 7.4, BSA 0.1%) containing 11.1 mM glucose was used to evaluate GIIS. Other islet groups were used to analyze the insulinotropic effects of the cholinergic agonist, carbachol (Cch, 10 *μ*M), and the inhibitory effects of the alpha-adrenergic agonist, oxymetazoline (Oxy, 10 *μ*M), in the presence of 11.1 mM glucose. This incubation occurred under the same conditions, gassed (CO_2_, 5%) at 37°C for 60 min. The resulting insulin produced was measured by the RIA technique. The resulting islets (100–150) were isolated and used for protein expression analysis. Briefly, after digestion with collagenase, the islets were cleaned and solubilized in a buffer containing 100 mM Tris pH 7.5, 10 mM/L sodium pyrophosphate, 100 mM of sodium fluoride, 10 mM/L EDTA, 10 mM sodium vanadate, 2 mM phenylmethylsulfonyl fluoride (PMSF), and 2% Triton X-100, pH 8.08.

The protein concentration in the supernatants was quantified by the Bradford assay [43]. For polyacrylamide and sodium dodecyl sulfate gel electrophoresis (SDS-PAGE) and Western blot analysis, samples were treated with Laemmli buffer. After heating at 95°C for 5 min, the proteins were separated by electrophoresis (30 *μ*g/lane, 10% gel) and transferred to nitrocellulose membranes. Membranes were blocked overnight at 4°C in Tris-buffered saline containing 5% skimmed milk. Subsequently, the membranes were incubated with polyclonal antibodies against OXPHOS (1:1000; ab110413-Abcam, Waltham, Massachusetts, United States) and IL-10 (1:1000; sc8438–Santa Cruz, Dallas, Texas, United States). After washing, the membranes were incubated for 1 h with a respective secondary antibody (1:10.000, Sigma), followed by exposure to an ImageQuant LAS 4000 Mini (GE Healthcare Bio-Sciences, Uppsala, Sweden) to detect chemiluminescence of the membranes from nitrocellulose. Ponceau was used as an internal control. Band intensities were quantified by the LabImage 1D software for gels and Western blots were analyzed using Kapelan Bio-Imaging solutions. Data are expressed as the normalization of the protein intensities and ponceau.

### 2.9. Morphometric Analysis and Collagen Deposition in Endocrine Pancreas

Immediately after euthanasia, the pancreas (*n* = 4 rats/group) was found, removed, cleaned, and immediately transferred to Alfac fixation solution (85 parts 70% ethanol, 10 parts formalin - containing 37% formaldehyde - and 5 parts glacial acetic acid) for 24 h. Subsequently, the organ was stored in alcohol (70%) until histological processing. For histological analysis, the organ was subjected to successive stages of dehydration, clarification in xylol, and embedded in Paraplast (McCormick Scientific, Leica Biosystems, Buffalo Grove, Illinois). The impregnated tissue was sectioned at 5 *μ*M using a Reichert Jung rotary microtome (Leica RM 2025 Microsystems Inc., Wetzlar, Germany) and stained with Mason's trichrome. Five slides were prepared for each pancreas, with two semiserial slices and an interval of 200 *μ*m. The slices were stained with Masson's trichrome and photographed at magnifications of 4×, 10×, 20×, and 40× using Olympus BX51 (Olympus Microscope, Shinjuku, Japan). Digital photographs were taken with a 36-bit, 1280 × 1024 pixel color digital camera with a DP71 controller (Olympus).

The number of islets in 10 semiserial sections was counted for each group using a microscope at 10× magnification; for comparison between groups, the average number of islets per section was used. Images from 104 to 133 islets per group were randomly selected to measure the area and area of collagen deposition inside the islet with the help of Image J 1.42q software (National Institutes of Health (NIH), Bethesda, Maryland), available from the NIH website (http://rsb.info.nih.gov/ij, accessed August 18, 2022). The islets were classified by area size into five categories: 400–599, 600–3999, 4000–7999, 8000–11,999, and 12,000–15,999 (*μ*m^2^). The collagen area was expressed as the percentage of islet total area present: less than 10% of the total islet area, between 10% and 20%, or more than 20%. The shape of the islets was classified as oval, round, and irregular, where the frequencies of shapes were used for analysis.

### 2.10. VN Activity Recording

At 150 days of life and after 12 h of fasting, 3–4 rats per Ob group were randomly selected and subjected to analysis of the VN, according to the technique described by Leon-Quinto and Magnan [44] adapted. Briefly, the rats were anesthetized with thiopental (45 mg/kg), and a small incision was made in the ventrocervical region of the neck. Then, the upper VN branch was located, cleaned, and recorded via the electronic system. The output of the neural signal was acquired by an Insight interface (Insight, Riberão Preto, SP, Brazil). After signal stabilization for 5–10 min, 20 record frames of 15 s from each animal were randomly chosen for spike counting. VN activity was analyzed for 5 s to determine the number of spikes. Peaks of greater than 0 mV were recorded as neural activity. The mean number of spikes was used as the nerve firing rate for each mouse (spikes/5 s).

### 2.11. Statistical Analysis

Data are expressed as mean and standard deviation (SD). Shapiro–Wilk's test was used to evaluate normality. Student's *t*-Test or Mann–Whitney *U* was performed to evaluate differences between CTL-SHAM and Ob-SHAM. The effects of surgery in the Ob groups were evaluated by two-way ANOVA, obtaining *p* values for vagotomy (*V*), splenectomy (*S*), and interaction (*I*, vagotomy vs. splenectomy). Significant *F* values were then analyzed in Tukey's post hoc test; when necessary, data were normalized. For BW evolution, food intake, ipGTT, and ipPTT were used for three-way repeated-measures ANOVA or in the mixed-effects model when values were missing. In this case, *F* represents the time (*T*) effect. Chi-square (*χ*^2^) test was used for qualitative analyses for histological data. The effect size for the significant *p* value (< 0.05) was calculated in ANOVA results using eta squared (*η*^2^), following the guidelines: *η*^2^ < 0.01 considered a small effect, ≈0.06 a medium effect, and > 0.14 a large effect. For Tukey's post hoc comparisons, Cohen's d (*d*) was used, with guidelines: *d* < 0.2 considered a small effect, *d* ≈ 0.5 a medium effect, and *d* > 0.8 a large effect. For chi-square results, Cramér's V (*V*) was used, with guidelines as follows: *V* < 0.1 for a small effect, 0.1–0.3 for a medium effect, and *V* > 0.3 for a large effect.

## 3. Results

### 3.1. Hypothalamic Obesity

Comparisons of adiposity, metabolism, and endocrine pancreas histology between hypothalamic Ob-SHAM and nonobese CTL-SHAM rats are shown in Supporting Information: Table S1 and Figure S1.

Despite having lower BW and NAL (*p* < 0.0001), Ob-SHAM rats presented higher values of LI (*p* < 0.0001) and augmented M-WAT (*p* = 0.0005) and I-WAT (*p* = 0.0055) depots, compared to CTL-SHAM animals. Moreover, the Ob-SHAM group presented normoglycemia (*p* = 0.4715), accompanied by fasting hyperinsulinemia (*p* = 0.0268), increased total cholesterol (*p* = 0.0050), and hypertriglyceridemia (*p* = 0.0120), compared to the CTL-SHAM group. Thus, the Ob-SHAM group presented IR with higher TyG values (*p* = 0.0412) than CTL-SHAM animals (Supporting Information 1: Table S1). The effects of the surgeries on the murinometric and metabolic parameters of animals with hypothalamic obesity are described in Table 1.

Histological analysis revealed significant alterations in the pancreas of the Ob-SHAM rats, compared to the CTL-SHAM. Notably, the pancreas of Ob-SHAM animals exhibited increased adipocyte infiltration (Supporting Information 2: Figure S1) and greater vacuolization. Furthermore, collagen deposition within the pancreatic islets was markedly higher in Ob-SHAM rats, as evidenced by a reduced proportion of islets with < 10% of collagen area (*χ*^2^ 7.31; DF 1; *p* = 0.0068; Table 2) and a greater prevalence of islet with collagen deposition between 10% and 20% (*χ*^2^ 12.07; DF 1; *p* = 0.0005; Table 2) and > 20% (*χ*^2^ 4.57; DF 1; *p* = 0.0325; Table 2), compared to CTL-SHAM animals (Supporting Information 3: Figure S2 and Table 2). Despite these changes, the number of islets by section in the pancreas was not affected by hypothalamic obesity, and the islet size distribution remained predominantly within the medium-to-small range (Table 2).

### 3.2. Effects of VN and Spleen Ablation on Biometric, Adiposity, and Metabolic Variables in Ob Animals

VN ablation induces gastric stasis, resulting in an elevated stomach weight, as shown here by the vagotomy effect (*F*_(1, 31)_ = 89.19; *p* < 0.0001; *η*^2^ = 0.71—large effect). Thus, Ob-VAG and Ob-VAG + SPL animals presented higher stomach weights, compared to the Ob-SHAM (*p* = 0.0001; *d* = 4.95—large effect) (*p* < 0.0011; *d* = 7.45—large effect) and versus Ob-SPL (*p* < 0.0001; *d* = 5.81—large effect) (*p* < 0.0001; *d* = 8.35—large effect) groups, respectively. Moreover, a significant *I* effect (*F*_(1, 31)_ = 6.664; *p* = 0.0148; *η*^2^ = 0.05—medium effect) was also noted for this parameter, where Ob-VAG + SPL rats had higher stomach weights than Ob-VAG animals (*p* = 0.0216; *d* = 3.07—large effect). In contrast, VN ablation did not modify spleen weight in Ob rats. At 150 days of life, the BW and NAL were not significantly altered by surgeries.

However, LI was influenced by vagotomy (*F*_(1, 32)_ = 8.433; *p* = 0.0066; *η*^2^ = 0.19—large effect). Thus, Ob-VAG rats presented reduced LI values compared to the Ob-SHAM group (*p* = 0.0345; *d* = 2.87—large effect). Vagotomy also influenced fat deposition in the visceral M-WAT depot (*F*_(1, 33)_ = 34.01; *p* < 0.0001; *η*^2^ = 0.50—large effect), and Ob-VAG rats demonstrated lower M-WAT weight when compared to the Ob-SHAM (*p* = 0.0058; *d* = −3.57—large effect) and Ob-SPL (*p* = 0.0008; *d* = −4.31—large effect) groups. Similarly, Ob-VAG + SPL had a lower M-WAT weight than the Ob-SHAM (*p* = 0.0020; *d* = −3.95—large effect) and Ob-SPL (*p* = 0.0003; *d* = −4.69—large effect) groups.

In contrast, vagotomy and splenectomy did not influence the subcutaneous I-WAT depot weight. Vagotomy slightly increased fasting glycemic levels (*F*_(1, 32)_ = 5.693; *p* = 0.0231; *η*^2^ = 0.26—large effect). Thus, the vagotomized groups demonstrated higher fasting glycemia than nonvagotomized animals (Ob-VAG vs. Ob-SHAM: *p* = 0.0335; *d* = 2.22—large effect; Ob-VAG + SPL vs. Ob-SHAM: *p* = 0.0488; *d* = 2.05—large effect; Ob-VAG versus Ob-SPL: *p* = 0.0134; *d* = 2.62—large effect; Ob-VAG + SPL vs. Ob-SPL: *p* = 0.0488, *d* = 2.47—large effect). Vagotomy modifies insulin circulating levels (*F*_(1, 29)_ = 13.22; *p* = 0.0011; *η*^2^ = 0.31—large effect) with Ob vagotomized groups presenting lower insulin plasma levels in comparison to the nonvagotomized animals (Ob-VAG vs. Ob-SHAM: *p* = 0.0097; *d* = −2.76—large effect; Ob-VAG + SPL vs. Ob-SHAM: *p* = 0.0366; *d* = −2.19—large effect; Ob-VAG vs. Ob-SPL: *p* = 0.0064; *d* = −2.94—large effect; Ob-VAG + SPL vs. Ob-SPL: *p* = 0.00247; *d* = −2.39—large effect). Neither vagotomy nor splenectomy changed plasma total cholesterol levels. However, vagotomy reduced plasma triglyceride levels (*F*_(1, 40)_ = 26.53; *p* < 0.0001; *η*^2^ = 0.36—large effect) with Ob-VAG (*p* = 0.0001; *d* = −4.85—large effect) and Ob-VAG + SPL (*p* < 0.0001; *d* = −5.08—large effect) groups presenting lower triglyceride levels than the Ob-SHAM animals. The TyG index, a marker of IR, was affected by vagotomy (*F*_(1, 35)_ = 10.05; *p* = 0.0032; *η*^2^ = 0.20—large effect). Thus, the Ob-VAG (*p* = 0.0143; *d* = −3.21—large effect) and Ob-VAG + SPL (*p* = 0.0211; *d* = −3.05—large effect) groups had lower TyG values than Ob-SHAM animals. The IL-10 circulating plasma levels were not altered either by vagotomy or splenectomy (Table 1).

### 3.3. Effects of Vagotomy and Splenectomy on Food Intake, BW, and FE

We found time effect on BW evolution (*F*_(10,400)_ = 213.1; *p* < 0.0001; *η*^2^ = 0.34—large effect) and food intake (*F*_(10,400)_ = 102.3; *p* < 0.0001; *η*^2^ = 0.57—large effect) during growth of Ob rats. Thus, while BW rises, the food intake was reduced over time; neither variable is significantly influenced by vagotomy or splenectomy (Supporting Information 3: Figure S2A and B). Similarly, vagotomy or splenectomy did not influence the BW gain (Supporting Information 3: Figure S2B) or FE (Supporting Information 3: Figure S2D).

### 3.4. Glucose and Pyruvate Tolerance in Obese Rats Submitted to Vagotomy and Splenectomy Surgeries

**Figure 1:**
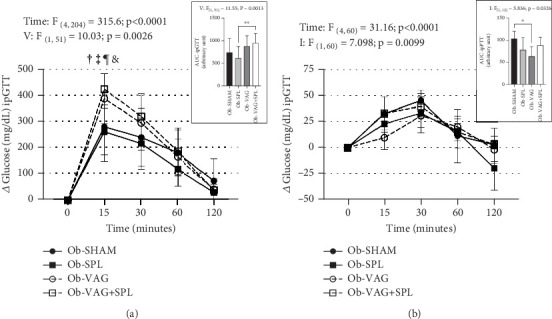
Glycemic curve in the glucose and pyruvate tolerance test. Data are means (SD); *n* = 14 rats/group for intraperitoneal glucose tolerance test (ipGTT), and *n* = 4 rats/group for intraperitoneal pyruvate tolerance test (ipPTT). Glycemic curves over time in ipGTT (a), on the right, respective area under the curve (AUC). Glycemic curves over time in ipPTT (b), on the right, respective area under the curve (AUC). Ob-SHAM: sham-operated obese group. Ob-SPL: splenectomized obese group. Ob-VAG: vagotomized obese group. Ob-VAG + SPL: vagotomized and splenectomized obese group. The time effect in three-way ANOVA is represented above the graph with *F*, as well as the vagotomy (*V*) effect and interaction (*I*) between vagotomy and splenectomy. Symbols are statistical differences between groups in ipGTT at 15 min: ^†^Ob-VAG and ^‡^Ob-VAG + SPL versus Ob-SHAM; ^¶^Ob-VAG versus Ob-SPL; ^&^Ob-VAG-SPL versus Ob-SPL; ⁣^∗^*p* value ≤ 0.05 and ⁣^∗∗^*p* value ≤ 0.01 in AUC, Tukey's post hoc test.

The effects of the absence of VN and spleen on the ipGTT are shown in Figure 1. After i.p. glucose load (2 g/kg), significant increases in blood glucose levels were seen at times 15, 30, and 60 min, compared to baseline and 120 min, reflecting time effects (*F*_(4,204)_ = 315.6; *p* < 0.0001; *η*^2^ = 0.78—large effect). Vagotomy influenced glucose levels during ipGTT (*F*_(1, 51)_ = 10.03; *p* = 0.0026; *η*^2^ = 0.02—small effect). Thus, at 15 min, the Ob-vagotomized animals showed increased blood glucose values, compared to nonvagotomized groups (Ob-VAG vs. Ob-SHAM: *p* = 0.0280; *d* = 3.76—large effect; Ob-VAG + SPL vs. Ob-SHAM: *p* = 0.0021; *d* = 5.03—large effect and Ob-VAG vs. Ob-SPL: *p* = 0.0034; *d* = 4.33—large effect; Ob-VAG + SPL vs. Ob-SPL: *p* < 0.0001; *d* = 5.58—large effect). In consequence, vagotomy effects (*F*_(1, 51)_ = 11.55; *p* = 0.0013; *η*^2^ = 0.17—large effect) were noted in the AUC and Ob-VAG + SPL rats had higher glucose AUC, compared to the Ob-SPL group (Figure 1 upper; *p* = 0.0062; *d* = 3.44—large effect). The ipPTT is shown in Figure 1. Increases in blood glucose levels were observed after the pyruvate load (1 g/kg), reflecting time effects (*F*_(4, 60)_ = 31.16; *p* < 0.0001; *η*^2^ = 0.59—large effect). Interactions, vagotomy versus splenectomy, influenced AUC of glucose during ipPTT (*F*_(1, 12)_ = 5.836; *p* = 0.0326; *η*^2^ = 0.29—large effect), with Ob-VAG rats showing lower glucose values than Ob-SHAM (Figure 1 above; *p* = 0.0196; *d* = −2.69—large effect), without affecting the Ob-VAG + SPL.

### 3.5. Effects of Vagotomy and Splenectomy Surgeries on VN Activity Rate in Obese Rats

**Figure 2:**
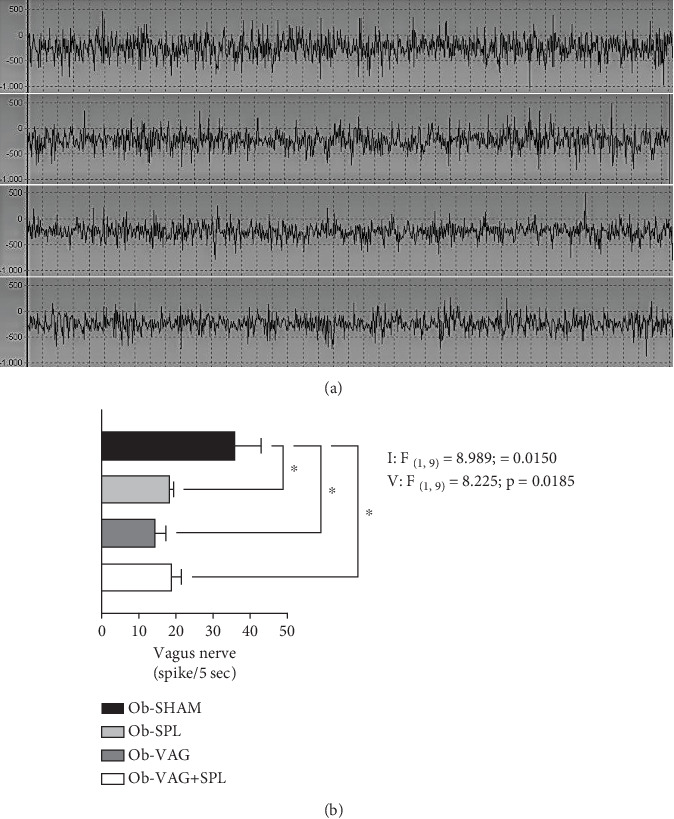
Recording of vagus nerve activity. Data are means (SD); *n* = 3–4 rats/group. Illustrative images of the frames that show the peaks of vagus nerve activity over time (a) and the graph of the number of firing peaks in 5 s/animal (b) (results were obtained randomly from 15-s frames). Ob-SHAM: sham-operated obese group. Ob-SPL: splenectomized obese group. Ob-VAG: vagotomized obese group. Ob-VAG + SPL: vagotomized and splenectomized obese group. (*V*) vagotomy effect and interaction (*I*) between vagotomy and splenectomy in two-way ANOVA are next to the graph with *F*. Statistical differences between groups were represented (⁣^∗^*p* value < 0.05, Tukey's post hoc test).

Interactions, vagotomy versus splenectomy (*F*_(1, 9)_ = 8.989; *p* = 0.0150; *η*^2^ = 0.31—large effect) and vagotomy (*F*_(1, 9)_ = 8.225; *p* = 0.0185; *η*^2^ = 0.29—large effect) effects, were observed on VN activity rate. Thus, post hoc tests saw fewer spikes in cervical VN for the Ob-SPL (*p* = 0.0295; *d* = −3.47—large effect), Ob-VAG (*p* = 0.0133; *d* = −4.01—large effect), and Ob-VAG + SPL (*p* = 0.0473; *d* = −3.15—large effect) groups, in relation to the VN activity rate of Ob-SHAM animals (Figure 2).

### 3.6. Effects of VN and Spleen Ablation on Morphometric and Collagen Deposition in the Endocrine Pancreas of Obese Animals

**Figure 3:**
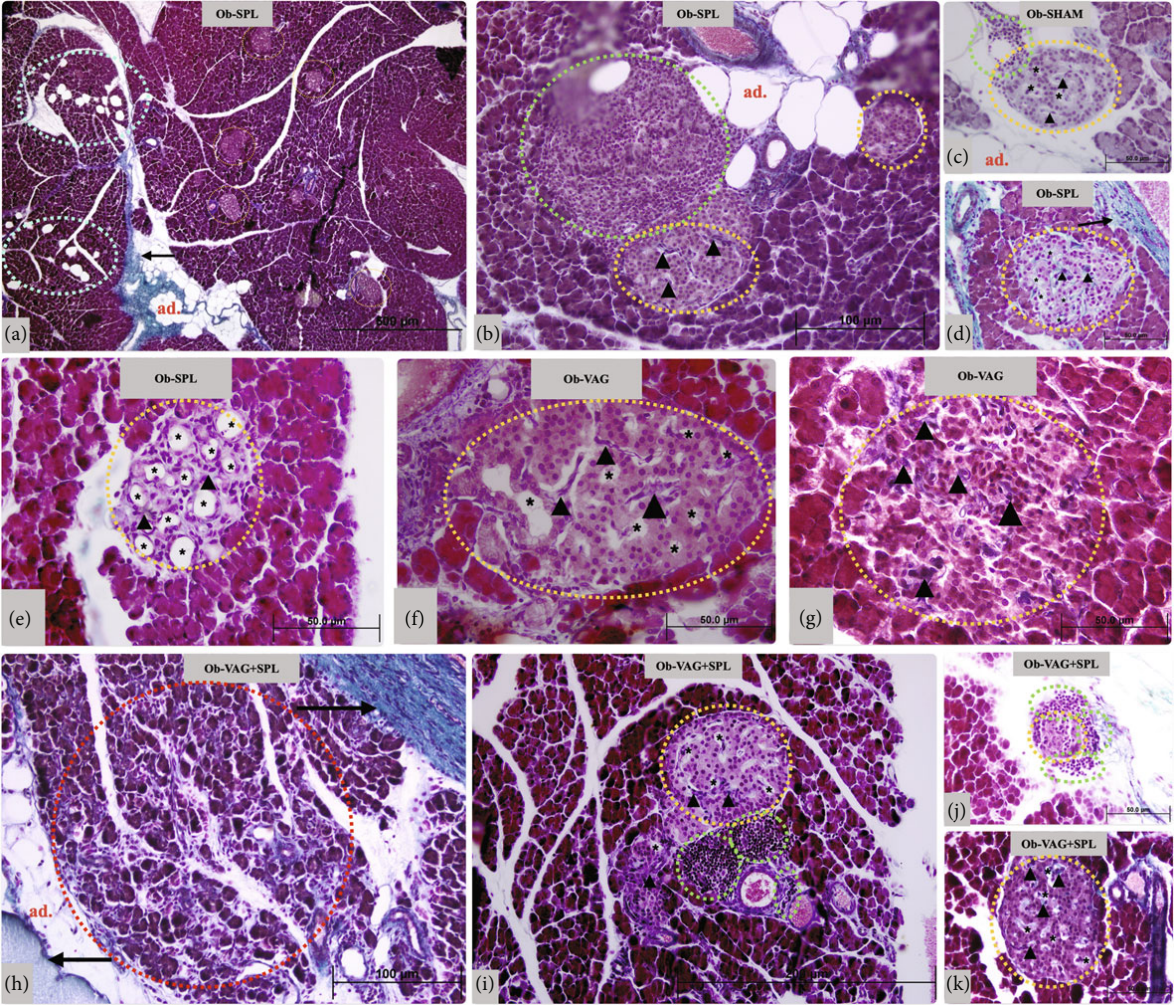
Islet morphology in obese rats submitted to VAG and/or SPL. Illustrative images of pancreatic sections stained with Masson's trichrome from obese (Ob.) groups, sham-operated (Ob-SHAM) (c), splenectomized (Ob-SPL) (a, b, d, e), vagotomized (Ob-VAG) (f, g), and vagotomized and splenectomized (Ob-VAG + SPL) (h–k); can be seen in the images: collagen deposition inside the islets (arrowhead); lipomatosis (ciano circle); adipocytes (ad.); leukocytes (green circle); vacuolization (∗); diffuse atrophy (red circle), interlobular septa (arrow). The scale bar is in the bottom right corner.

Representative photomicrographs showing the central histopathological appearance in the pancreas of obese animals submitted to vagotomy and splenectomy surgeries are shown in Figure 3. The morphological analysis is presented in Table 2.

The pancreas of Ob-VAG rats exhibited reduced adipocyte infiltration compared to the Ob-SHAM rats, with no notable alterations in interlobular septa. In this group, some islets displayed discrete vacuolization (Figure 3f; 40×; scale bar, 50 *μ*m), similar to that observed in Ob-SHAM islets. However, the islets of Ob-VAG rats showed more pronounced cellular disorganization (Figure 3g; 40×; scale bar, 50 *μ*m), characterized by altered islet morphology, with fewer round islets (*χ*^2^ 5.23; DF 1; *p* = 0.0222; *V* = 0.28—medium effect; Table 2) and a greater number of irregular-shaped islets (*χ*^2^ 4.24; DF 1; *p* = 0.0393; *V* = 0.31—medium effect; Table 2) compared to Ob-SHAM rats. The percentage of collagen deposition within the islets, normalized by islet area, was higher in vagotomized groups. Ob-VAG islets exhibited a reduced number of islets with < 10% collagen deposition (*χ*^2^ 10.64; DF 1; *p* = 0.001; *V* = 0.37—medium effect; Table 2) and an increased number of islets with > 20% collagen area (*χ*^2^ 4.23; DF 1; *p* = 0.0396; *V* = 0.35—medium effect; Table 2), relative to Ob-SHAM islets (arrowhead; Figure 3f,g, scale bar, 50 *μ*m).

The pancreas of Ob-SPL rats exhibited large adipocytes (ad. in orange; Figure 3a; 4×; scale bar, 500 *μ*m) and thickened fibrotic tissue in the interlobular region (black arrow; Figure 3a; 4×; scale bar, 500 *μ*m). Notably, the acinar parenchyma displayed lipomatosis (ciano circles, Figure 3a), a feature absent in all other experimental groups. Additionally, Ob-SPL animals showed increased leukocyte infiltration around to islet (green circle, Figure 3b, 10×; scale bar, 100 *μ*m) and the presence of larger vacuoles (∗, Figure 3b; 40×; scale bar, 50 *μ*m) compared to the pancreas of Ob-SHAM rats (Figure 3c, 40×; scale bar, 50 *μ*m). Compared to Ob-SHAM rats, islets of Ob-SPL animals exhibited more irregular-shaped islets (*χ*^2^ 10.56; DF 1; *p* = 0.0011; *V* = 0.40—medium effect) and a greater number of very small islets (*χ*^2^ 4.5; DF 1; *p* = 0.0339; *V* = 0.37—medium effect). Collagen deposition within the islets did not differ between Ob-SPL rats and Ob-SHAM groups.

Histological analysis of the pancreas in the Ob-VAG + SPL group revealed reduced adipocyte infiltration, with thickening of fibrotic tissue in the interlobular septa and periductal region (black arrow; Figure 3h; 10×; scale bar, 100 *μ*m). Additionally, diffuse atrophy of acinar parenchyma was observed in this group (red circle; Figure 3h; 10×; scale bar, 100 *μ*m). Notably, the islets of Ob-VAG + SPL rats exhibited similar leukocyte infiltration to that seen in the Ob-SPL green (green circle; Figure 3i, 10×, scale bar 200 *μ*m; Table 2). Moreover, numerous islets displayed vacuolization and pronounced cellular disorganization (Figure 3j; 40×, scale bar, 50 *μ*m). Islets from Ob-VAG + SPL rats had a more irregular shape (*χ*^2^ 4.24; DF 1; *p* = 0.01393; *V* = 0.28—medium effect Table 2) than islets of the Ob-SHAM animals. Collagen deposition (arrowhead; Figure 3k; 40×, scale bar, 50 *μ*m) was not different between Ob-VAG + SPL animals and Ob-SHAM group (Table 2).

The absence of VN or spleen did not alter islet numbers in the endocrine pancreas of Ob-SHAM rats. However, an interaction (vagotomy vs. splenectomy) effect was observed for this variable (*F*_(1, 12)_ = 4.911; *p* = 0.0468; *η*^2^ = 0.27—large effect), although no significant differences were detected in the post hoc test (Table 2).

### 3.7. Insulin Secretion, OXPHOS, and IL-10 Expression in Islets From Obese Animals Submitted to VN and Spleen Ablation

**Figure 4:**
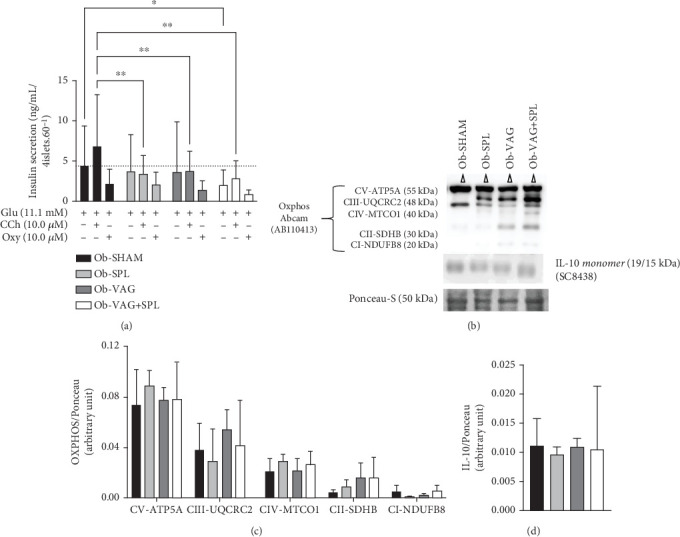
Glucose, cholinergic, and adrenergic effects, OXPHOS, and interleukin-10 (IL-10) expression in isolated pancreatic islets from obese rats submitted to VAG and/or SPL. Data are means (SD); *n* = 4–6 rats per group. Glucose (Glu) induced insulin secretion (GIIS) for 11.1 mM glucose (a); insulinotropic effect induced by carbachol (Cch) (a), and inhibitory effect of oxymetazoline (Oxy) (a). Protein expression of mitochondrial complex OXPHOS (c) and interleukin-10 (IL-10) (d) and illustrative Western blot bands (b). Ob-SHAM: sham-operated obese group. Ob-SPL: splenectomized obese group. Ob-VAG: vagotomized obese group. Ob-VAG + SPL: vagotomized and splenectomized obese group. Statistical differences between groups in two-way ANOVA were represented by ⁣^∗^*p* value ≤ 0.05 and ⁣^∗∗^*p* value ≤ 0.01, Tukey's post hoc test.

The association of vagotomy and splenectomy resulted in a reduction of GIIS in isolated pancreatic islets. Thus, islets isolated from the Ob-VAG + SPL group showed lower GIIS than Ob-SHAM rats (*p* = 0.00296; *d* = −2.18—large effect) (Figure 4a). This response was not associated with changes in the expression of electron transport chain proteins, as shown by OXPHOS data (Figure 4b,c). Similarly, the vagotomy and splenectomy surgeries did not modify IL-10 protein expression in the islets of Ob rats (Figure 4d).

Surgeries influenced the Cch-insulinotropic response in islets from Ob animals, where pancreatic islets from the Ob-SPL (*p* = 0.0028; *d* = −3.01—large effect), Ob-VAG (*p* = 0.061; *d* = −2.76—large effect), and Ob-VAG + SPL (*p* = 0.0024; *d* = −3.06—large effect) groups showed a reduced response to Cch, when compared to islets from Ob-SHAM rats. In contrast, neither VN nor spleen absence significantly altered the inhibitory effect of the alpha-adrenergic agonist, Oxy, on islets of Ob animals (Figure 4a).

## 4. Discussion

In the present study, we confirmed that neonatal treatment with MSG leads to obesity and prediabetic features in adult rats, such as hyperinsulinemia, dyslipidemia, and IR, as previously demonstrated by us [26, 34] and other researchers [45, 46]. For the first time, we have shown that the islets of obese hypothalamic rats exhibit greater collagen deposition than those of nonobese animals. It is well established that fibrosis results in islet dysfunctions and may be linked to the pathogenesis of Type 2 diabetes mellitus (T2DM). Elevated glucose, insulin, cytokines, and angiotensin II seem to be associated with fibrosis in islets from obese rodents [47, 48].

Afferent and efferent signals from the VN can modulate metabolism and inflammatory responses, and vagal dysfunctions may be linked to metabolic immune abnormalities commonly found in obese states [8]. Hypervagal activity and sympathetic hypofunction are associated with high adiposity, dyslipidemia, and islet dysfunction in obese hypothalamic rodents [12, 17]. In this context, early VN ablation (at 30 days of life) reduces adiposity, normalizes insulin levels, and prevents disturbances in glucose homeostasis in adult rats with hypothalamic obesity [12, 17]. Accordingly, we demonstrated that vagotomy at 60 days of life effectively reduced visceral WAT depots, reestablishing triglyceride profiles and insulin sensitivity even after 90 days after intervention, without altering food intake or FE.

We have previously shown that the presence of the spleen is necessary for the effects of vagotomy on adiposity and metabolism in the nonobese state [34]. However, in this study, the antiadiposity and fasting metabolic benefits of vagotomy were maintained in splenectomized obese rats, indicating changes in the interactions between the VN and the spleen in hypothalamic obesity.

The VN is also a well-recognized modulator of hepatic gluconeogenesis. In the healthy state, surgical resection of the hepatic branch of the VN reduces the inhibitory effect of this pathway on gluconeogenesis by half, leading to a marked increase in liver glucose production [49]. In contrast, in the present study, Ob-VAG rats presented lower gluconeogenesis after pyruvate overload, suggesting altered VN control in the gluconeogenesis of Ob animals.

Ob-hypothalamic rats exhibit pyruvate intolerance, a condition linked to elevated hepatic glucose flux and hypertriglyceridemia [46, 50]. The capacity of insulin to inhibit hepatic glucose production is compromised in individuals with increased intrahepatic triglycerides [51]. Our data demonstrate that, following VN cutting, Ob rats experienced a reduction in triglycerides and an improvement in IR; these changes may contribute to better gluconeogenesis regulation. Supporting these findings, Ob-hypothalamic rats treated with the PPAR*α*/*γ* dual agonist, chiglitazar, exhibited lower triglyceride levels, enhanced insulin sensitivity, and improved hepatic gluconeogenesis [46]. Therefore, for the first time, we provide evidence that vagotomy surgery can prevent pyruvate intolerance in Ob-hypothalamic rats.

The VN serves as a vital link between the central nervous system and peripheral organs, playing a critical role in glucose homeostasis and immune responses through both afferent and efferent fibers [52]. Interestingly, about 80% of VN fibers are afferent, while the remaining 20% are efferent [53]. Selective stimulation of the efferent VN fibers results in an increase in insulinemia. In contrast, when stimulation is applied only to the afferent cervical VN fibers, or in both pathways, it leads to hyperglycemia in healthy anesthetized rats, without a corresponding rise in insulinemia. This suggests that the afferent signals of the VN provide negative feedback that inhibits insulin secretion [54]. However, this does not rule out the possibility that cervical VN stimulation may enhance hepatic gluconeogenesis mediated by the sympathetic system when VN stimulation is chronic [55]. Further research is needed to clarify this intricate dynamic of autonomic control in the context of obesity and diabetes.

In the present study, we performed total subdiaphragmatic vagotomy and noted a significantly reduced cervical VN firing rate 90 days postsurgery, likely reflecting interruptions in autonomic afferent and efferent signals. In the obese-hypothalamic rats, disruptions in glucose homeostasis have been linked to vagal hyperactivity [17]; however, the individual contributions of afferent and efferent vagal signals to glucose homeostasis in this obese model remain unknown. Our data show that VN cutting slightly raises fasting glucose levels and improves pyruvate tolerance; glucagon levels influence both processes, and as mentioned earlier, obese-hypothalamic rats exhibit alterations in glucagon regulation [50].

Several studies indicate that VN and the spleen interact to modulate immune and metabolic responses [7, 19, 22]. Our research group has demonstrated that the interactions between VN and the spleen can influence adiposity and insulin secretion [28, 34]. In this study, we show that the absence of the spleen alters the effects of vagotomy on glucose and pyruvate tolerance in Ob-hypothalamic rats. Thus, Ob-SPL rats exhibit better glucose tolerance compared to the Ob-VAG + SPL animals. Furthermore, lacking a spleen counteracts the beneficial effects of vagotomy on pyruvate tolerance, suggesting that the interplay between the spleen and VN could regulate gluconeogenesis in the liver of Ob-hypothalamic animals. Accordingly, we previously established the spleen's influence on glycemic control in Ob-hypothalamic animals [26, 27].

Moreover, our current work has also demonstrated that spleen ablation reduces cervical VN rate activity in Ob-hypothalamic animals, suggesting that signals derived from splenic tissue could be involved in vagal hyperactivity in this model of obesity. During the anti-inflammatory vagal reflex, afferent vagal fibers are activated by several cytokines, including IL-1*β* [52]. The spleen is an important source of cytokines during the inflammatory process [56]. In healthy rats, we have shown that the spleen modulates IL-1*β* [28]. Therefore, it is possible that the absence of the spleen alters circulating cytokine levels in obese hypothalamic rats, influencing the firing rate of the VN and affecting glycemic control. Further studies are necessary to confirm this hypothesis. The anti-inflammatory, spleen-derived IL-10 has been proposed as a protective factor for the liver and endocrine pancreas in high-fat obese rats [24]. We have demonstrated that the VN can modulate plasma IL-10 levels in nonobese rats [28], but not in obese hypothalamic rats [34].

In the current study, neither the absence of the spleen nor the cutting of the VN significantly altered plasma IL-10 levels or pancreatic IL-10 protein expression in Ob-hypothalamic rats. This suggests that the anti-inflammatory vagal reflex does not modulate IL-10 in this obese model. Interestingly, the splanchnic nerve supply to the adrenal gland is linked to the anti-inflammatory vagal reflex and splenic IL-10 production [57], an effect that relies on adrenaline and *β*-adrenergic receptors. Ob-hypothalamic rats exhibit reduced catecholamine secretion [58] and lower sympathetic tone; these conditions could explain the absence of the effects of vagotomy and splenectomy on IL-10 levels in this obese model.

Pancreatic islets are richly innervated by VN terminals [59], and cholinergic signaling within the islets is crucial for regulating insulin secretion and maintaining glucose homeostasis [60]. Furthermore, VN also influences beta cell proliferation [61]. Ob-hypothalamic rats exhibit vagal hyperactivity [12, 32], islet hypertrophy, and insulin hypersecretion, while early vagotomy reverses these abnormalities in the endocrine pancreas [12, 17, 31].

In this study, a significant reduction in GIIS was observed only in the islets of the Ob-VAG + SPL animals and was not associated with changes in the area or number of islets. It should be noted that, in this study, vagotomy was performed at 60 days of life, and as previously reported, Ach can modulate beta cell mass at an earlier age [62, 63]. We have demonstrated that splenectomy can reduce GIIS in pancreatic islets from Ob-hypothalamic rats [26, 27]. Similar vagotomy also reduces insulin hypersecretion in this obese model [17].

In addition, we noted that when the combined ablation of the VN and spleen was performed, a reduction in insulinotropic cholinergic responsiveness was observed in islets from Ob-hypothalamic rats. As a potentiator, the cholinergic insulinotropic action depends on glucose's stimulatory effects [64]. Therefore, it is likely that the diminished cholinergic responsiveness is due to a reduced GIIS also observed in the islets from Ob-VAG + SPL rats. Collectively, our results indicate that vagal and splenic-derived factors contribute to insulin hypersecretion in islets from Ob-hypothalamic rats, likely by modulating GIIS as well as insulinotropic cholinergic responsiveness.

The balance of collagen deposition in islets is crucial for proper GIIS [65]. Ach serves as the primary neurotransmitter of the VN, and several studies have highlighted its role in regulating collagen deposition in organs such as the liver, kidney, and heart [66–68]. Our findings suggest that vagotomy increases collagen deposition within islets, indicating a link between pancreatic VN activity and collagen deposition in the endocrine pancreas of Ob-hypothalamic rats. In a pancreatic beta cell line, Type I and V collagen are important for stimulating insulin secretion [69, 70]. The nicotinic Ach receptor in the pancreas participates in collagen deposition [71]. However, it remains unclear how the VN and Ach influence collagen deposition within the islet and the relationship between this process and GIIS in the islets of Ob-hypothalamic rats.

Pancreatic fatty infiltration is associated with obesity and T2DM [72]. Interestingly, we also observed that VN ablation reduces adipocyte infiltration in the endocrine pancreas of Ob-hypothalamic rats. Accordingly, adipocyte infiltration can be related to autonomic neural activity in the endocrine pancreas [73].

In healthy and high-fat diet–induced obesity in rodents, splenectomy increased inflammation and collagen infiltration within the islets due to a response mediated by a reduction in IL-10 [24]. In contrast, the present study found that islets from Ob-SPL rats did not exhibit increased collagen deposition or any changes in IL-10 protein expression. However, we observed that the absence of the spleen exacerbates adverse effects on pancreatic structure; notably, Ob-splenectomized animals showed increased adipocyte infiltration, heightened inflammatory processes, and vacuolization. Thus, it seems that splenic-derived factors are crucial for maintaining islet structure in Ob-hypothalamic rats.

Augmented lipid accumulation in the pancreatic parenchyma has been previously reported in aging rodent models and is associated with diabetes [74]. Moreover, peri-insulitis, which is characterized by increased leukocyte infiltration, is observed during the initial phase of morphological changes in a nonobese Type 1 diabetes model and contributes to beta cell destruction [75, 76]. Therefore, the increased vacuolization processes observed in islets from Ob-SPL rats in the present study may indicate an imbalance between apoptotic and proliferative factors caused by inflammation, oxidative stress, or hormonal factors, leading to the loss of beta cells.

Immunological pancreatic splenic effects are well recognized, particularly in diabetes. Splenic B cells can modulate islet inflammation; thus, autoreactive splenic B cells may migrate to the pancreas where they infiltrate the islets and are restimulated by islet beta cell autoantigens [77]. Islets from animals with T2DM show increased activated proinflammatory infiltrated macrophages in spleen tissue [78].

In the Ob-VAG + SPL rats, we observed increased leukocyte infiltration in the endocrine pancreas, suggesting that the absence of the spleen and VN worsens inflammation in this organ. According to the vagal anti-inflammatory hypothesis, the VN reduces the inflammatory response under healthy conditions, thereby modulating splenic functions [79]. As previously noted, the spleen and *α*7nAChR-mediated signaling are crucial to the anti-inflammatory effects of the VN in a healthy state [80]. Activation of the *α*7nAChR has been shown to delay the clinical onset of diabetes and decrease inflammation in the NOD mouse model [81]. Therefore, it is likely that the absence of the spleen results in diminished Ach signaling to the pancreas of splenectomized rats, heightening the susceptibility of this organ to lesions, as indicated by our histopathological analysis. This hypothesis is supported by our data showing that spleen ablation reduces VN activity, suggesting that signals arising from splenic tissue may regulate VN firing to the endocrine pancreas.

The present study has several limitations that we highlight: (i) we performed total subdiaphragmatic transection, cutting the afferent and efferent vagal fibers, which makes it impossible to evaluate the individual contributions of these distinct afferent or efferent arms to metabolic or immune responses; (ii) important cytokines, such as TNF-*α*, and hormones, like glucagon, known for their significant impact on glycemic control, were not measured; and (iii) the *α*7nAChR protein content was not assessed in either islets or splenocytes. Further studies should be conducted to clarify these points.

## 5. Conclusion

Total subdiaphragmatic vagotomy reduces WAT content; improves hypertriglyceridemia, IR, and pyruvate intolerance; and modulates adipocytes and collagen deposition in the pancreas of obese hypothalamic male rats. However, spleen-derived signals appear to be important to vagotomy's effects on gluconeogenesis, influencing VN rate firing, insulin secretion, and pancreas histology. This suggests that the splenic–vagal axis participates in metabolic disturbances induced by hypothalamic obesity.

## Figures and Tables

**Table 1:** Effects of vagotomy (VAG) and/or splenectomy (SPL) on biometric, adiposity, plasma metabolic parameters, and insulin resistance (IR) in obese rats at 150 days of life.

**Parameters**	**Ob**				**(** **p** ** value) two-way ANOVA**		
	**SHAM**	**SPL**	**VAG**	**VAG + SPL**	**I**	**V**	**S**
BW (g)	295.50 (42.97)	289.10 (27.37)	282.30 (41.91)	275.60 (30.71)	0.9595	0.1801	0.6126
NAL (cm)	19.44 (0.73)	19.70 (1.03)	20.10 (1.15)	19.67 (1.20)	0.3251	0.3730	0.8078
LI	0.344 (0.007)	0.337 (0.009)	0.329 (0.014)^**†**^	0.331 (0.011)	0.4918	**0.0066**	0.2143
M-WAT (g/100 g BW)	2.26 (0.62)	2.36 (0.40)	1.46 (0.35)^**†**¶^	1.35 ± (0.50)^**‡**&^	0.4907	**< 0.0001**	0.9948
I-WAT (g/100 g BW)	0.51 (0.20)	0.57 (0.22)	0.57 (0.26)	0.58 (0.16)	0.7114	0.5914	0.6637
Stomach (g/100 g BW)	0.49 (0.07)	0.46 (0.04)	0.85 (0.18)^**†**¶^	1.08 (0.24)^**‡**&*Ψ*^	**0.0148**	**< 0.0001**	0.0740
Spleen (g/100 g BW)	0.103 (0.007)	—	0.100 (0.009)	—	—	0.3323	—
Glycemia (mg/dL)	83.18 (17.25)	80.63 (17.19)	97.40 (4.27)^**†**¶^	95.62 (8.25)^**‡**&^	0.9312	**0.0024**	0.6260
Insulinemia (ng/mL)	0.58 (0.26)	0.59 (0.09)	0.30 (0.17)^**†**¶^	0.37 (0.21)^**‡**&^	0.7413	**0.0011**	0.5653
Total cholesterol (mg/dL)	157.00 (34.72)	144.22 (31.98)	154.00 (51.59)	136.44 (25.42)	0.8463	0.6622	0.2233
Triglycerides (mg/dL)	397.73 (142.72)	284.32 (90.61)	192.75 (69.22)^**†**^	193.40 (72.71)^**‡**^	0.0540	**< 0.0001**	0.0566
TyG	9.63 (0.37)	9.23 (0.54)	8.92 (0.48)^**†**^	8.96 (0.48)^**‡**^	0.1600	**0.0032**	0.2326
IL-10 (pg/mL)	36.85 (13.38)	41.95 (16.64)	42.73 (20.32)	48.80 (21.56)	0.9551	0.4644	0.5206

*Note:* Data are mean (SD), *n* = 8–16 rats/group. IL-10 *n* = 4–5 rats/group. ANOVA two-way test was performed, and *p* value < 0.05 was bold, column shows *F* surgery effects to *I* (interaction), *V* (vagotomy), and *S* (splenectomy). LI: Lee index ([3√BW (gram)/NAL (centimeter)]). TyG: [Ln(fasting triglycerides) (mg/dL) × fasting glucose (mg/dL)/2]. Symbols mean *p* < 0.05 in Tukey post hoc test.

Abbreviations: BW, body weight; cm, centimeters; g, grams; g/100 g BW, grams per hundred grams of body weight; IL-10, interleukin-10; I-WAT, inguinal white adipose tissue; mg/dL, milligrams per deciliter; M-WAT, mesenteric white adipose tissue; NAL, nasoanal length; ng/dL, nanograms per deciliter; Ob-SHAM, sham-operated obese group; Ob-SPL, splenectomized obese group; Ob-VAG, obese group, vagotomized; Ob-VAG + SPL, obese group, vagotomized and splenectomized; pg/mL, picograms per milliliter.

^**§**^Ob-SPL.

^**†**^Ob-VAG versus Ob-SHAM.

^**‡**^Ob-VAG + SPL versus Ob-SHAM.

^¶^Ob-VAG versus Ob-SPL.

^&^Ob-VAG-SPL versus Ob-SPL.

^*Ψ*^Ob-VAG-SPL versus Ob-VAG.

**Table 2:** Morphological analysis in the pancreas from control and obese groups and effects of vagotomy and splenectomy.

**Alteration**	**CTL-SHAM**	**Ob-SHAM**	**Ob-SPL**	**Ob-VAG**	**Ob-VAG + SPL**				
Lipomatosis	−	−	+	−	−				
Leukocyte infiltrate	−	−	++	−	++				
Vacuolization	+	++	+++	++	++				

**Shape**	**Relative frequency (%)**					**(** **p** ** value) chi-square**			
						∗	**§**	**†**	**‡**
Oval	38	41	33	43	35	0.6586	0.9146	0.8312	0.8272
Round	44	42	33	24	35	0.8330	1.0000	**0.0222**	0.7448
Irregular	18	18	34	33	29	1.0000	**0.0011**	**0.0393**	**0.0393**

**Islet area (*μ*m** ^**2**^ **)**									
400–599	4	9	16	5	8	0.1088	**0.0339**	0.1967	0.8185
600–3999	56	53	44	55	54	0.8514	0.7796	0.9250	0.5210
4000–7999	20	27	22	27	22	0.2579	0.8964	0.8946	0.6858
8000–11,999	15	8	13	9	9	0.1615	0.1166	1.0000	0.6547
12,000–15,999	5	2	4	5	6	0.2568	0.2568	0.2568	0.0955

**% collagen area/islet**									
< 10	83	51	56	24	36	**0.0068**	0.0771	**0.0011**	0.2207
> 10–< 20	14	39	31	54	46	**0.0005**	1.0000	0.1277	0.2158
> 20	3	10	14	22	18	**0.0325**	0.1936	**0.0396**	0.0770
						**(** **p** ** value)**	**Two-way ANOVA**		
**t** **-test**	**I**	**V**	**S**
Islet number/section	29 (8)	15 (12)	24 (5)	27(9)	18 (4)	0.0702	**0.0468**	0.4376	0.9403

*Note:* Data was obtained by 104–116 islets/group. − means absence and +, ++, +++ mean presence in three different intensities. Bold number means *p* < 0.05. The islet number per section was obtained by ten semiserial cut section of the pancreas, and the average of number of islets per section (SD) was used.

In the chi-square test, symbols represent the statistical difference between Ob-SHAM and CTL-SHAM (⁣^∗^), Ob-SPL (**§**), Ob-VAG (**†**), and Ob-VAG + SPL (**‡**).

## Data Availability

The data supporting the findings of this study are available from the corresponding author upon reasonable request.

## Supporting Information

Supporting Information 1 Table S1 presents the biometric, metabolic, and islet morphology changes induced by hypothalamic lesions as a consequence of MSG neonatal treatment. Despite having lower BW and NAL (*p* < 0.0001), Ob-SHAM rats presented higher values of LI (*p* < 0.0001) and augmented M-WAT (*p* = 0.0005) and I-WAT (*p* = 0.0055) depots, compared to CTL-SHAM animals. Moreover, the Ob-SHAM group presented normoglycemia (*p* = 0.4715), accompanied by fasting hyperinsulinemia (*p* = 0.0268), increased total cholesterol (*p* = 0.0050), and hypertriglyceridemia (*p* = 0.0120), compared to the CTL-SHAM group. Thus, the Ob-SHAM group presented IR with higher TyG values (*p* = 0.0412) than CTL-SHAM animals. This characteristic reproduces the main alteration of the model of hypothalamic obesity, reproduced by us and other groups.

Supporting Information 2 Histological changes are seen in Table S1 (down panel) and Figure S1, which has the pancreas illustrative photomicrographs of the Ob-SHAM group and CTL-SHAM. It can be noted that the pancreas of Ob-SHAM demonstrated higher adipocyte infiltration and vacuolization, in comparison to the pancreas of the CTL-SHAM groups. Moreover, the percentage of collagen deposition inside the islets was higher in islets from Ob-SHAM rats than CTL-SHAM animals (Figure S1 and Table 1). The number or area of islets in the pancreas was not influenced by hypothalamic obesity.

Supporting Information 3 Figure S2 presents the effects of surgeries (VAG and/or SPL) on food intake and body weight gain, allowing us to calculate the FE of these animals, and we do not observe any alteration in these variables after the intervention.
