# Supplementary material for: Vagal Splenic-Dependent Effects Influence Glucose Homeostasis, Insulin Secretion, and Histopathology of the Endocrine Pancreas in Hypothalamic Obese Male Rats: Vagus Nerve and Spleen Interactions Affect the Endocrine Pancreas
Source: ScientificWorldJournal. 2025 Apr 17;2025:9910997. doi: 10.1155/tswj/9910997 (PMC12021492; doi:10.1155/tswj/9910997)
Supplement: Supporting Information 1 — Table S1 presents the biometric, metabolic, and islet morphology changes induced by hypothalamic lesions as a consequence of MSG neonatal treatment. Despite having lower BW and NAL (p < 0.0001), Ob-SHAM rats presented higher values of LI (p < 0.0001) and augmented M-WAT (p = 0.0005) and I-WAT (p = 0.0055) depots, compared to CTL-SHAM animals. Moreover, the Ob-SHAM group presented normoglycemia (p = 0.4715), accompanied by fasting hyperinsulinemia (p = 0.0268), increased total cholesterol (p = 0.0050), and hypertriglyceridemia (p = 0.0120), compared to the CTL-SHAM group. Thus, the Ob-SHAM group presented IR with higher TyG values (p = 0.0412) than CTL-SHAM animals. This characteristic reproduces the main alteration of the model of hypothalamic obesity, reproduced by us and other groups. [file 9910997.f1.pdf]

Table S1 – Biometric, metabolic effects and morphology of pancreas islets of hypothalamic lesions induced by MSG at 150 days of life

	CTL-SHAM	Ob-SHAM	p-value
**BW (g)**	422.40 (34.89)	295.50 (42.97)	<0.0001#
**NAL (cm)**	23.16 (0.39)	19.44 (0.73)	<0.0001#
**LI (g/mm³)**	0.32 (0.01)	0.34 (0.01)	<0.0001*
**M-WAT (g/100g BW)**	0.97 (0.17)	2.26 (0.62)	0.0005*
**I-WAT (g/100g BW)**	0.24 (0.09)	0.51 (0.19)	0.0055*
**Glycemia (mg/dL)**	89.70 (23.28)	86.46 (18.90)	0.7454
**Insulinemia (ng/mL)**	0.32 (0.18)	0.58 (0.26)	0.0268*
**Total Cholesterol (mg/dL)**	76.40 (60.08)	157.00 (34.72)	0.0050#
**Triglycerides (mg/dL)**	247.20 (70.78)	397.70 (142.70)	0.0120*
**TyG**	9.20 (0.45)	9.63 (0.337)	0.0412*
**Islet (number/section)**	29.80 (8.11)	15.26 (12.36)	0.0702
**Islet area (µm²)**	4539.49 (3848.84)	3852.38 (3606.75)	0.1784
**Collagen deposition (% collagen/islet total area)**	6.70 (4.81)	11.45 (6.75)	<0.0001#

Data are means (SD), n= 8-16. p-value was considered significant ≤0.05, statistical differences in #: Test Mann-Whitney U. *: Student's T-test. CTL: control. Ob: obese. SHAM: sham operation. BW: body weight. NAL: naso-anal length. LI: Lee Index ([$\sqrt[3]{BW(}$ (g)/NAL (cm)]). M-WAT: mesenteric white adipose tissue. I-WAT: inguinal white adipose tissue. TyG: [Ln(fasting triglycerides) (mg/dl) x fasting glucose (mg/dl)/2]. g: grams. cm: centimeters. g/100g BW: grams per hundred grams of body weight. mg/dL: milligrams per deciliter. ng/mL: nanograms per milliliter. µm²: square micrometer.
