# Supplementary material for: Vagal Splenic-Dependent Effects Influence Glucose Homeostasis, Insulin Secretion, and Histopathology of the Endocrine Pancreas in Hypothalamic Obese Male Rats: Vagus Nerve and Spleen Interactions Affect the Endocrine Pancreas
Source: ScientificWorldJournal. 2025 Apr 17;2025:9910997. doi: 10.1155/tswj/9910997 (PMC12021492; doi:10.1155/tswj/9910997)
Supplement: Supporting Information 2 — Histological changes are seen in Table S1 (down panel) and Figure S1, which has the pancreas illustrative photomicrographs of the Ob-SHAM group and CTL-SHAM. It can be noted that the pancreas of Ob-SHAM demonstrated higher adipocyte infiltration and vacuolization, in comparison to the pancreas of the CTL-SHAM groups. Moreover, the percentage of collagen deposition inside the islets was higher in islets from Ob-SHAM rats than CTL-SHAM animals (Figure S1 and Table 1). The number or area of islets in the pancreas was not influenced by hypothalamic obesity. [file 9910997.f2.pdf]

Supplementary Figure S1. Islets morphology in obese and control rats

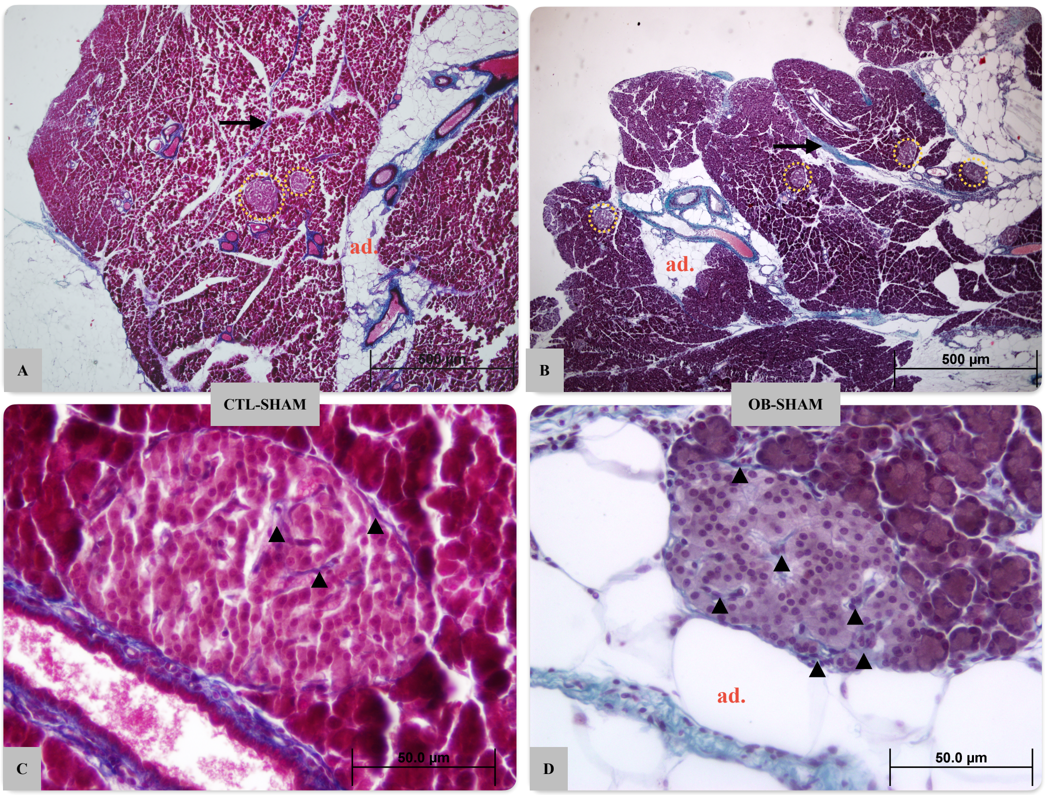

Illustrative images of pancreatic sections stained with Masson's Trichrome: Control (CTL) animal, with simulated operation (SHAM), 4x objective, scale bar, 500µm (A) and magnified image of the islet of this group at 40x objective, scale bar, 50µm (C). Obese animal (Ob) with simulated operation (SHAM), 4x objective, scale bar 500µm (B) and magnified image of the islet of this group at 40x objective; scale bar, 50µm (D). Adipocytes (ad.) were seen in both groups in the region of the interlobular septa and moderate thickening of fibrotic tissue was seen in Ob-SHAM (arrow, B). The islets (yellow circles) mostly kept their normal shape. Collagen was found in the peri-islet region and inside the islets (arrowhead).
