# Supplementary material for: Vagal Splenic-Dependent Effects Influence Glucose Homeostasis, Insulin Secretion, and Histopathology of the Endocrine Pancreas in Hypothalamic Obese Male Rats: Vagus Nerve and Spleen Interactions Affect the Endocrine Pancreas
Source: ScientificWorldJournal. 2025 Apr 17;2025:9910997. doi: 10.1155/tswj/9910997 (PMC12021492; doi:10.1155/tswj/9910997)
Supplement: Supporting Information 3 — Figure S2 presents the effects of surgeries (VAG and/or SPL) on food intake and body weight gain, allowing us to calculate the FE of these animals, and we do not observe any alteration in these variables after the intervention. [file 9910997.f3.pdf]

**Supplementary Figure S2. Effeccts of VAG and/or SPL surgeries on Body Weight (BW), food intake, and feed efficiency in Ob rats at 150 days of life**

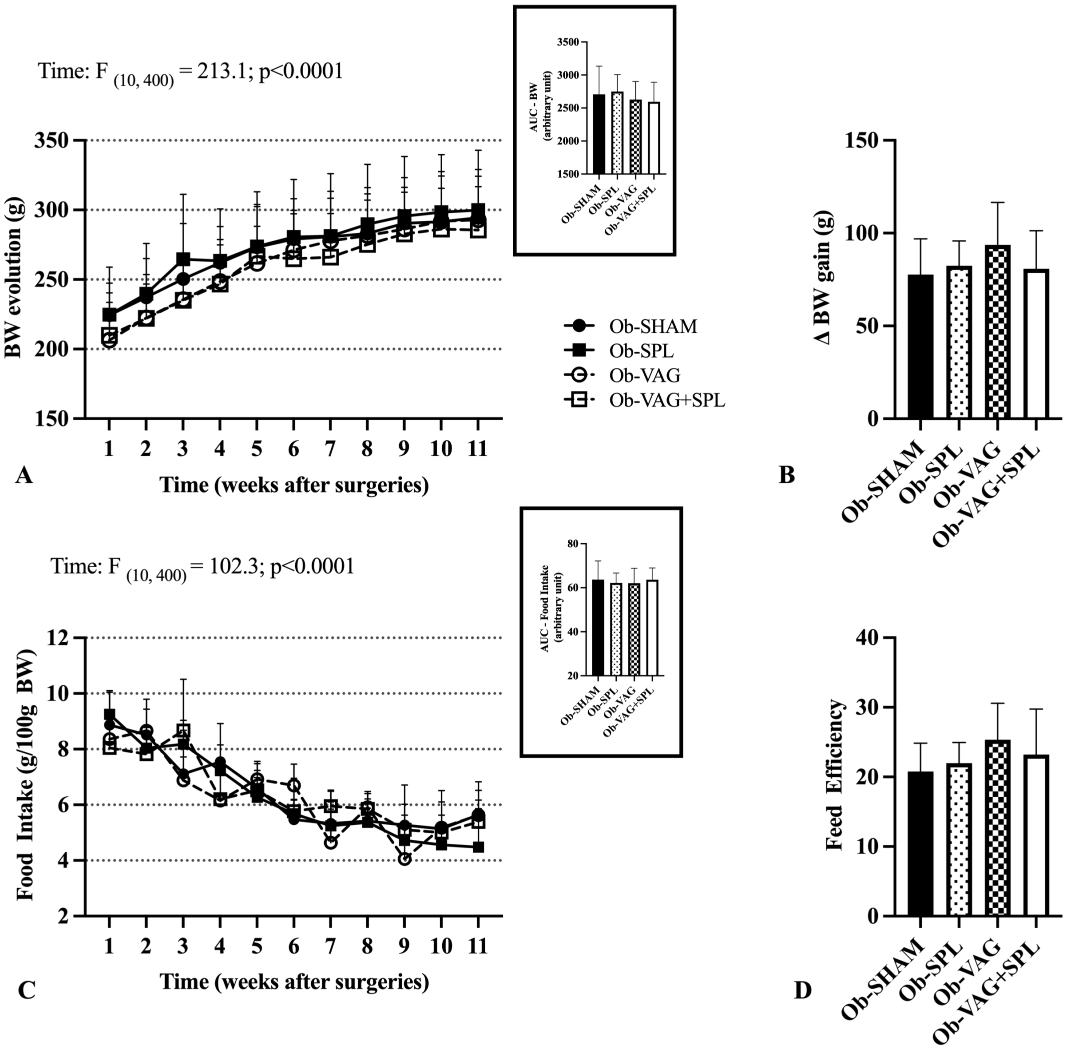


Data are means (SD); n= 11 rats/group. BW evolution over time after surgeries (A) and Food intake in the same period (C); AUC of BW evolution (A, above) and AUC of food intake (C, above); BW gain (Δ = final-initial weight) (B) and feed efficiency (Δ body weight (g)/∑ food intake (g)*100) (D). Ob-SHAM: sham-operated obese group. Ob-SPL: splenectomized obese group. Ob-VAG: vagotomized obese group. Ob-VAG+SPL: vagotomized and splenectomized obese group. BW: Body Weight. g: grams. The time effect in Three-way ANOVA is represented above the graph with F.
